# Does Radiofrequency Ablation Add to Chemotherapy for Unresectable Liver Metastases?

**DOI:** 10.1007/s11888-012-0122-9

**Published:** 2012-03-17

**Authors:** Klaas M. Govaert, Charlotte S. van Kessel, Martijn Lolkema, Theo J. M. Ruers, Inne H. M. Borel Rinkes

**Affiliations:** 1 Department of Surgery, University Medical Centre Utrecht, Heidelberglaan 100, 3584CX Utrecht, the Netherlands; 2 Department of Medical Oncology, University Medical Centre Utrecht, Heidelberglaan 100, 3584CX Utrecht, the Netherlands; 3 Department of Surgery, The Netherlands Cancer Institute/Antoni van Leeuwenhoek Hospital, Plesmanlaan 121, 1066CX Amsterdam, the Netherlands; 4 Department of Surgery, University Medical Center Utrecht, Room G04-228, PO Box 85500, 3508 GA Utrecht, The Netherlands

**Keywords:** Colorectal liver metastases, Radio frequency ablation, Chemotherapy, Local recurrence, Overall survival, Progression free survival, Combination therapy

## Abstract

In patients with unresectable colorectal liver metastases (CRLM), radiofrequency ablation (RFA) might be a good alternative, whenever possible. In contrast to systemic therapy, the aim of RFA is to achieve complete local tumor control in an attempt to provide long-term survival. In this article we discuss the available evidence regarding the treatment of patients with unresectable CRLM, focusing on RFA in conjunction with modern systemic therapies. We observed that the available evidence in the existing literature is limited, and often consists of level 2 and 3 evidence, thereby hampering any firm conclusions. Nonetheless, RFA seems superior to chemotherapy alone in patients with liver-only disease amenable for RFA. However, the combination of RFA and chemotherapy has been demonstrated to be feasible and safe, lending support to the concept of RFA followed by chemotherapy, in order to reduce local recurrence rates and prolong survival.

## Introduction

One in two colorectal cancer patients will develop liver metastases at some point during their disease [1, 2]. In recent years significant progress has been made in the treatment of metastatic colorectal cancer. Large randomized clinical trials have been conducted showing a beneficiary effect of different first-line and second-line chemotherapy regimens such as capecitabine, oxaliplatin, and irinotecan, with or without the vascular-endothelial growth factor inhibitor bevacizumab [3, 4]. Also, there is no doubt that third-line treatment with epidermal growth factor inhibitors like cetuximab and panitumumab results in prolonged survival [5–7]. Due to these regimens the median survival of patients with metastatic colorectal cancer has improved from 6 months [8] to more than 2 years [3, 4, 8–15].

The only potentially curative option for patients with colorectal liver metastases (CRLM) is surgical resection. Five-year survival probabilities of up to 50% can be achieved [16, 17•, 18]. Multimodality treatment including the use of neoadjuvant chemotherapy has resulted in resectability rates of 20–25% [19–21]. The remaining 75–80% of CRLM patients are not eligible for resection, because of the anatomy making resection impossible, due to extensive intrahepatic metastatic lesions, or the presence of unresectable extensive extrahepatic disease (EHD). Without treatment these patients have a marginal survival, with only 0–2% being alive after 5 years [22, 23].

During the last decade treatment options for patients with unresectable CRLM have evolved markedly, especially in the era of systemic treatment and local ablative therapies. Of those local ablative techniques, radiofrequency ablation (RFA) is most widely used [24, 25]. In contrast to systemic therapy, the aim of RFA is to achieve complete local tumor control in an attempt to provide long-term survival. However, the role of RFA in unresectable CRLM is still under debate as there are limited data comparing progression-free survival and overall survival in unresectable CRLM patients between systemic treatment and RFA. In this article we will discuss the available evidence regarding the treatment of patients with unresectable CRLM, focusing on RFA in conjunction with modern systemic therapies.

## Radiofrequency Ablation

In the last decade of the 20th century, RFA was initially described as a procedure to treat hepatocellular carcinoma; subsequently it was introduced as a treatment for unresectable CRLM [26–29]. Many studies have been conducted to assess the effect of RFA on progression-free survival (PFS) and overall survival (OS) in patients with unresectable CRLM. Reported 3-year survival rates vary between 37% and 77% [30••, 31–33], while 5-year survival rates range between 27% and 36% [30••, 33]. These data involve patients with unresectable CRLM without EHD treated with open RFA. For percutaneous or laparoscopic RFA the outcomes appear to be far less favorable [30••].

The major problems with RFA are the high rates of intrahepatic and local disease recurrence. This is caused by either the existence of intrahepatic micrometastases, or incomplete tumor destruction by RFA, respectively [34]. Several studies that were conducted to identify liver recurrence patterns after RFA for CRLM showed intrahepatic recurrence rates (new lesions) varying between 32% and 62.5% [30••, 35–38]. Reported local recurrence rates (at site of RFA) also vary widely, resulting in recurrence rates of 1.6% up to 60% [24, 30••, 36, 39, 40]. It is important, however, to make a distinction between lesion-based and patient-based analyses, as the later results in higher recurrence rates [41, 42].

The great variety in reported data is illustrated in Table 1, revealing extensive heterogeneity in study populations, procedures, and design. In addition, several studies have included both CRLM and HCC, regardless of the entirely different biological background and behavior of these entities. Needless to say, comparisons of (disease free) survival and recurrence rates should be viewed with great caution. Tumor size is associated with local recurrence; the larger the tumor the higher the probability of incomplete ablation and, hence, local recurrence. Although newer RFA probes have resulted in more effective ablation with larger ablation regions, nowadays tumor size ≤3 cm is considered most favorable to achieve compete tumor destruction [43, 44]. However, tumor size >3 cm is not an absolute contraindication as effective ablation of larger zones can be obtained by using multiple probes [45, 46].

**Table 1:** Overview of key studies assessing recurrence patterns and survival following RFA: this table illustrates the wide variety in study design and results

Author	Year of publication	Study type	Level of evidence	RFA approach	No. of pts	No. of patients with EHD	Lesion no. (median)	Lesion size (median)	Median OS (months)	Median DFS (months)	3 years	5 years	Local recurrence
Gillams [57•]	2009	PCS	2b	P	309	105	3	3.5	27	NR	NR	NR	NR
Veltri [69]	2008	PCS	2b	O/P	122	25	1.6	2.9	31.5	8.6	38.0%	22.0%	26.3%
Abitabile [40]	2007	PCS	2b	O/L/P	47	0	3.1	2	39	27.7% at 33 months	57.0%	21.0%	8.8%
Sorensen [70]^a^	2007	PCS	2b	O/P	102	0	3.3	2.2	32	NR	46.0%	26.0% (4 year)	NR
Siperstein [25]	2007	PCS	2b	L	234	55	2.8 (mean)	3.9 (mean)	24	6	20.2%	18.4%	NR
Machi [58]	2006	PCS	2b	O/L/P	100	NR	3.5 (mean)	3.0 (mean)	28	13	42.0%	31.0%	6.7%
Jakobs [71]	2006	PCS	2b	P	68	11	2.7	2.3	NR	NR	68.0%	NR	NR
van Duijnhoven [49]	2006	PCS	2b	O/P	87	0	2	2.9	27.8 (mean)	15 (mean)	NR	NR	37.0%
Berber [53]	2005	PCS	2b	L	135	40	3.2	4.1	28.9	6^b^	NR	NR	?
Abdalla [31]	2004	PCS	2b	O	57	0	1	2.5	NR	4.4% at 36 months	37.0%	22.0% (4 year)	9.0%
Solbiati [72]	2001	RCS	3b	P	109	NR	1.6	NR	30	12	33.0%	NR	29.6%

*L* laparoscopic; *NR* not reported; *O* open; *P* percutaneous; *PCS* prospective cohort study; *RCS* retrospective cohort study

^a^Sorensen: 86% percutaneous procedures, 14% open surgical procedure with/without resection

^b^6 months in patients without EHD

Furthermore, RFA of lesions close to large vessels has been questioned as the so called “heat sink phenomenon” by the blood flow in the vessel passing the probe results in less effective heat buildup, thereby preventing complete tumor destruction. A retrospective study by Lu et al. actually demonstrated that lesions in contiguity of vessels larger than 3 mm is a strong independent predictor of incomplete tumor destruction as recurrence occurred more frequently in perivascular lesions compared to non-perivascular lesions (48% vs 7% respectively) [47]. Perivascular localization remained an independent predictor during multivariable analysis, although the study was underpowered to draw firm conclusions. Moreover, the heat sink effect can be reduced by intermittent clamping of the involved vessel [48, 49]. Therefore, tumor localization near vessels should not be considered an absolute contraindication for RFA.

Another important factor determining local recurrence following RFA is the type of procedure that is applied: open, laparoscopic, or percutaneous. Plausibly, the latter involving the fewest procedural risks and the open approach being the most injurious one. So far, no randomized trials comparing the open surgical approach with the percutaneous approach have been conducted. Studies assessing the percutaneous approach often included patients with solitary or limited number of metastases, while studies assessing the open surgical approach often included a higher number of (larger) metastases per patient. The currently available evidence regarding survival demonstrates superior outcomes following the open approach compared to the laparoscopic and percutaneous procedures [24, 30••, 50–52]. Also, local recurrence rates are lower in the open surgical group compared with the percutaneous group (6–16% [31, 32, 39, 50] versus 17–59% [50, 53, 54], respectively). However, in the absence of data demonstrating improved survival outcomes when comparing similar groups of patients, the benefits of improved local control observed with the open approach must be weighed against the morbidity and cost associated with it . In addition, from the perspective that chemotherapy in combination with RFA debulking is reasonable, the risk of leaving behind a small number of tumor cells with the percutaneous approach may likely not be clinically detrimental in most cases in which this occurs. Moreover, if the lesion location is such that a local recurrence can be salvaged by subsequent resection, percutaneous RFA would seem reasonable provided that state-of-the-art CT guidance is available.

A noteworthy benefit of RFA is the possibility to perform repeated procedures. Several prospective cohort studies have shown that repeat procedures in patients with early (local or intrahepatic) recurrence after RFA results in similar survival rates as patients treated with RFA without intrahepatic recurrences [41, 49, 55•]. No doubt that introduction of percutaneous RFA application has widened the possibilities for repeated RFA. Therefore, each patient showing disease recurrence should be carefully evaluated for this option [30••, 55•].

So far, RFA has only been proposed as a potentially curative treatment option for selected patients with CRLM without EHD [56••]. However, the group of Siperstein and Berber published two prospective cohort studies (234 [54 with EHD] patients and 135 [40 with EHD] patients, respectively) investigating the role of RFA in patients with EHD [25, 53]. Siperstein et al. observed similar median OS in patients with and without EHD at the time of RFA (20 vs 25 months, respectively, NS). Berber et al. performed a multivariable analysis which showed no negative effect of EHD on survival. Moreover, they argued that in 70% of the deceased patients death was contributed to their intrahepatic disease progression. Hence, it was proposed that debulking of liver metastases by RFA in patients with EHD might be a helpful strategy. However, we believe that the presence of EHD in both studies did not result in a statistically significant negative effect (maybe due to underpowered study designs), and cannot be interpreted as results in favor to perform RFA in patients with EHD. In contrast, two studies by Gillams et al. and Machi et al. prospectively analyzed 309 and 100 patients with unresectable CRLM, respectively, in order to assess the influence of concomitant EHD on survival in patients with CRLM treated with RFA. Both studies found a significant negative effect on survival compared with patients without EHD (*P* < 0.05), especially for EHD other than lung metastases [57•, 58]. Based on the available evidence RFA should not be advised in patients with concomitant EHD.

Taken together, there is accumulating evidence that RFA of CRLM can result in long-term survival in selected cases. Optimal results may be achieved with an open surgical approach or CT-guided percutaneous procedures, in patients without EHD, in lesions <3 cm, while the procedure should be performed by experienced physicians [56••].

## RFA, Chemotherapy, or Combined Treatment

The debate is still ongoing on whether patients benefit most from RFA, systemic treatment, or a combination of the two. Reported OS following open RFA appears to be superior to that of chemotherapy (median overall survival 30–31 vs 20–23 months, respectively) [9, 32, 59•, 60]. However, several issues need to be addressed. First, most studies on RFA were conducted in patients with unresectable metastases confined to the liver, while all chemotherapy studies investigated patients with systemic disease, often including extensive intrahepatic disease and EHD. Also, other baseline characteristics such as tumor size and number differed between the RFA studies and the systemic treatment studies. Furthermore, several RFA studies combined data of patients with metastases from different primary sites. Moreover, some of the RFA studies included patients receiving RFA alone as well as patients who received a combination of RFA with either pre-treatment or post-treatment chemotherapy, thus hampering interpretation of the data due to induced heterogeneity.

In one of the rare comparative studies published hitherto, Ruers et al. investigated RFA versus systemic therapy in 201 patients harboring CRLM without EHD initially scheduled for surgery [32]. During laparotomy patients were allocated to one of three treatment arms: resection if feasible, local ablation therapy (sometimes combined with resection if resection [alone] was not feasible), or systemic treatment if neither of these procedures were possible. Baseline characteristics between the local ablation and systemic therapy groups were similar. Five-year OS was not significantly different between both groups, with 27% of the patients still alive in the local ablation group and 15% in the chemotherapy group. Median OS and DFS were 31 and 9 versus 26 and 0 months, respectively. If this is truly a significant difference in median overall survival, the difference is quite small and it would require substantially larger patient cohorts to confirm significance. Furthermore, the observed median OS of 26 months in the chemotherapy group is rather high when compared with other chemotherapy trials, which might be explained by the predominant inclusion of patients with CRLM confined to the liver. Abdalla et al. observed a significant (7 months) survival benefit for RFA compared to systemic therapy in CRLM patients [31]. This finding was based on data from consecutive series of 418 selected patients identified in their prospectively assembled database. Although the survival curves were converging at almost 55 months of follow up, 3-year survival rates were almost twice as high for patients receiving RFA compared to patients receiving chemotherapy. Thus, with RFA, the majority of people live longer compared to patients receiving chemotherapy, but eventually the survival curves of both groups will approach zero. However, important to mention is that the allocation to the different treatment groups was not random. Rather, it was determined by the extent of tumor burden determined intraoperatively, meaning the chemotherapy patients most likely had a higher tumor load.

Nonetheless, as the major problem regarding RFA involves the high rate of disease recurrence, it seems reasonable to seek for a combination treatment of RFA with systemic therapy. Further ground for such combination comes from preclinical studies where micrometastases surrounding the ablated region were recently shown to be triggered toward a more aggressive growth pattern following RFA so that combination with systemic therapy and/or hypoxia-activated prodrugs appears justified [61, 62]. Such combination strategies could be envisioned in two ways: 1) debulking tumor mass by RFA to reduce the tumor load needed to treat with systemic therapy, or 2) downstaging with systemic therapy followed by RFA of the remaining lesions. Literature regarding this topic is surprisingly scarce. Knudsen et al. evaluated the long-term survival of 36 patients treated with RFA for initially unresectable CRLM down-staged by systemic chemotherapy [63]. Patients with EHD were excluded from this study. They observed a median OS of 39 months with a 5-year survival rate of 34%. One should note that this study selected patients based on their responsiveness to systemic therapy, thereby introducing significant bias. Nonetheless, the reported 5-year disease-free survival of 14% is substantial.

Siperstein et al. conducted a prospective cohort study, in which 234 consecutive patients undergoing 292 RFA procedures for unresectable CRLM were evaluated [25]. The majority of these patients received systemic therapy prior to RFA and of those patients, 80% were poor or non-responders. In these patients median OS following RFA was 24 months, or 32 months from time of diagnoses as proposed by the authors. This is in sharp contrast to the 12 months from time of diagnosis for patients who are unresponsive to systemic therapy and *not* subsequently treated by RFA [53]. As the authors based this control group on data from 1999, it may be more justified to compare their data with the more recent chemotherapy studies mentioned earlier, which described an OS of up to 22.6 months [9, 64, 65]. Even though randomized data are non-existent, this would still represent a major improvement, with a 10-month benefit for patients receiving RFA following chemotherapy. Based on this study it would appear that RFA might have a role in controlling local disease for patients with unresectable CRLM who proved unresponsive to chemotherapy.

Regarding the question whether there is a role for tumor debulking by RFA followed by systemic treatment, data are limited. In 2007, Frezza et al. published a pilot study on 11 patients who underwent “debulking” by RFA of liver metastases of different origin prior to chemotherapy. With a mean follow-up of 22 months, none of these patients died from their disease while only two developed tumor recurrence [66]. In the aforementioned study by Siperstein et al., a significantly better OS of 28 months was observed in patients receiving chemotherapy following RFA, compared to 19 months for RFA alone [25]. However, as this was a purely observational finding and not the primary focus of the study, the authors were justifiably cautious to draw any conclusions. They argued that the observed effect might have been caused by selection bias by reserving “adjuvant” chemotherapy for patients responsive to RFA. Due to the limited number of patients and retrospective nature of the study, it is impossible to draw any firm conclusions from these studies.

The only prospective randomized trial assessing this topic is the CLOCC trial [67••]. In this randomized phase II trial 119 patients with <10 unresectable liver metastases without EHD were randomly assigned to either systemic treatment, or the combination of RFA followed by systemic treatment. In the chemotherapy-alone group 30 months OS was 57.6% compared to 61.7% in the combination group. However, this difference did not reach statistical significance, which may in part be explained by the much higher than expected observed survival data for patients treated with chemotherapy alone. Median PFS, however, was significantly different between both study arms. Median PFS in the combined treatment group was 16.8 months compared to 9.9 months in the chemotherapy-alone arm. As the randomized phase II design of the study was underpowered to detect differences in OS, the authors concluded that RFA plus systemic treatment results in significant benefit in PFS without significant benefit in OS at 30 months, and that longer follow-up should be awaited. Meanwhile, one cannot ignore a 30 months OS of 61.7% as an excellent result which, so far, has not been demonstrated in other studies. Taken together, the best available evidence points toward a benefit for the combination strategy using RFA and chemotherapy, although convincing proof is still lacking.

In an attempt to delineate whether the timing of chemotherapy treatment (pre-RFA or post-RFA) affects patient outcome, Sgouros et al. recently reported a prospective study comparing the use of RFA before or after chemotherapy in patients with unresectable CRLM [68]. Patients received either FOLFIRI before RFA, or FOLFOX or FOLFIRI post-RFA. Unfortunately, the total of included patients was low (*n* = 31). Moreover, patients were not randomized but allocated to one of the treatment schemes on the basis of tumor size, with patients harboring larger tumors starting with chemotherapy. In addition, 50% of the patients starting with chemotherapy did not receive RFA due to disease progression. Although PFS and OS did not differ significantly between the groups (13 and 21 months vs 10 and 21 for the pre-RFA and post-RFA groups, respectively), the study limitations render any firm conclusions problematic.

Apart from survival rates, a comparison *can* be made between RFA and systemic therapy regarding toxicity profiles and quality of life. Ruers et al. showed that patients receiving chemotherapy following laparotomy demonstrated a significantly lower quality of life compared to RFA following laparotomy [32]. Moreover, as disease-free periods can be achieved with RFA, patients may be provided time without toxicity. This is in sharp contrast to patients undergoing repetitive sessions of systemic therapy.

## Conclusions

The level of evidence emanating from the existing literature hampers any firm conclusions. Nonetheless, it would appear that RFA may prove superior to chemo alone in patients with liver-only disease amenable for this modality. Also, the combination of RFA and chemo has been demonstrated to be feasible and safe, lending support to the concept of RFA, whenever possible followed by chemotherapy.
